# Examining the Carbon Footprint of Orthopaedic Outpatient Care and Comparing the Emissions From In-Person Versus Virtual Models of Care: A Systematic Review

**DOI:** 10.7759/cureus.100475

**Published:** 2025-12-31

**Authors:** Lucy Lee-Smith, Joseph Andrews

**Affiliations:** 1 Trauma and Orthopaedics, Bangor Hospital, Bangor, GBR; 2 Law, Bangor University, Bangor, GBR

**Keywords:** carbon footprint, environmental sustainability, net zero, orthopaedic surgery, orthopaedic telemedicine, telemedicine, virtual clinic

## Abstract

Healthcare systems are responsible for a significant proportion of global greenhouse gas emissions. Trauma and orthopaedics has an exceptionally high emission profile in comparison to other specialities. This systematic review synthesises the available literature regarding the impact of virtual care/telemedicine on the carbon footprint of outpatient orthopaedics. Nine studies, encompassing over 500,000 patients, were included following a search across major databases. Virtual care has consistently been shown to have a smaller carbon footprint than traditional in-person appointments, primarily by reducing patient travel, resulting in a median reduction of 11.8 kgCO₂e per patient interaction. Secondary benefits, such as patient cost savings and improved accessibility, were also observed. However, the certainty of evidence was low due to the risk of bias across the studies; future research should aim to directly measure patient travel and other associated emissions.

## Introduction and background

Sustainability has gained interest in healthcare, with a strong intent to transition to greener practices [1]. The United Kingdom’s National Health Service has pledged to become the first carbon net-zero healthcare system by 2040 [2]. This evolution is crucial, as global healthcare systems contribute substantially to climate change, accounting for approximately 5% of global greenhouse gas (GHG) emissions [3].

Trauma and orthopaedics is one of the top three highest-emitting specialities [4]. This is partially due to the high volume of patients requiring regular follow-up, imaging, and examinations, which often involve travelling long distances. Notably, 7% of healthcare system emissions are derived from transportation [5]. A solution to this could be the adoption of virtual care or telemedicine, the delivery of patient care using technology, as conducting an outpatient review remotely negates the need for patient travel.

Telemedicine has been shown to lower the carbon footprint of healthcare, demonstrated in a 2024 systematic review [6]. The safety and financial advantages of virtual orthopaedic outpatient care are also well-supported by research [7]. To ascertain how telemedicine affects the carbon footprint of outpatient orthopaedic care, in particular, this systematic review synthesises currently available data in this field.

## Review

Methods

A systematic review was conducted in accordance with the standards described in the Cochrane Handbook and the Preferred Reporting Items for Systematic Reviews and Meta-Analyses (PRISMA) guidelines [8,9]. It is registered in the International Prospective Register of Systematic Reviews (PROSPERO) under Protocol No. CRD420251132499.

Search Strategy and Study Selection

Systematic searches were conducted from 27th to 29th September 2025 across multiple databases, including PubMed, Scopus, Ovid (MEDLINE and Embase), CINAHL, and Google Scholar, using specific PICO boolean strings (Appendices), as described in Table 1.

**Table 1:** PICO components for the systematic review research question PICO: population, intervention, comparators, and outcomes; kgCO₂e: kilograms of carbon dioxide equivalent, LCA: life cycle assessment, GHG: greenhouse gas

PICO research question	
Population	All patients attending orthopaedic outpatient services
Intervention	Virtual outpatient care
Comparators	In-person or hybrid outpatient care
Outcomes	Carbon footprint/GHG emissions/kgCO_2_e/lifecycle assessment/LCA

We included peer-reviewed studies published in English from 2008 onwards that examined patients attending orthopaedic outpatient services. Eligible studies evaluated virtual outpatient care (including telemedicine) compared with in-person or hybrid outpatient care. They reported empirical data on environmental outcomes, such as carbon footprint, GHG emissions, kgCO₂e, or life cycle assessment, including patient travel-related emissions. The post-2008 timeframe was applied to ensure technological relevance and comparability of sustainability outcomes. Studies were excluded if they focused on non-orthopaedic specialities or inpatient care, did not report emissions data or other environmental outcomes, or were published as reviews, editorials, or conference abstracts/posters without full empirical data. Publications not available in English and those published prior to 2008 were also excluded.

Due to the high volume of records retrieved from Google Scholar, only the first 300 results sorted by relevance were screened. Google Scholar was used as a supplementary search platform to identify peer-reviewed studies that may not yet be indexed or easily retrievable through traditional bibliographic databases. Search strings are detailed in the Appendices section.

Search results were imported into a reference manager, Zotero, and a systematic review platform, Rayyan, for practicality. The screening process was conducted in two distinct stages. Initially, titles and abstracts were reviewed against the predetermined inclusion criteria, followed by a full-text screening of potentially relevant articles. All screening and subsequent data extraction were carried out independently by two reviewers to ensure accuracy, and any discrepancies or ambiguities were settled through discussion to reach a consensus.

Data Extraction and Synthesis

Relevant data was extracted and recorded in a standardised form. Key data included study details (author, year, journal, country, design), population (setting, subspeciality, sample size), and intervention details (virtual platform, comparator, conversion methods). They reported outcomes (emissions in kgCO₂e, energy use, travel data and assumptions, and cost). To facilitate comparison, emission outcomes were converted to reductions in kgCO₂e per patient interaction and mean patient travel in miles.

The final list of eligible studies was assessed for risk of bias using established tools. The Risk of Bias in Non-randomised Studies of Interventions (ROBINS-I) tool was used for all non-randomised studies, and the Cochrane Risk of Bias 2 (RoB 2) tool was applied to the sole randomised controlled trial (RCT) [10,11]. The Grading of Recommendations Assessment, Development and Evaluation (GRADE) tool was used to rate the certainty of the evidence synthesised [12].

Due to heterogeneity in methodologies and modelling assumptions, a random-effects meta-analysis was not feasible. A narrative synthesis was performed to summarise and interpret the findings of the literature.

Results

**Figure 1:**
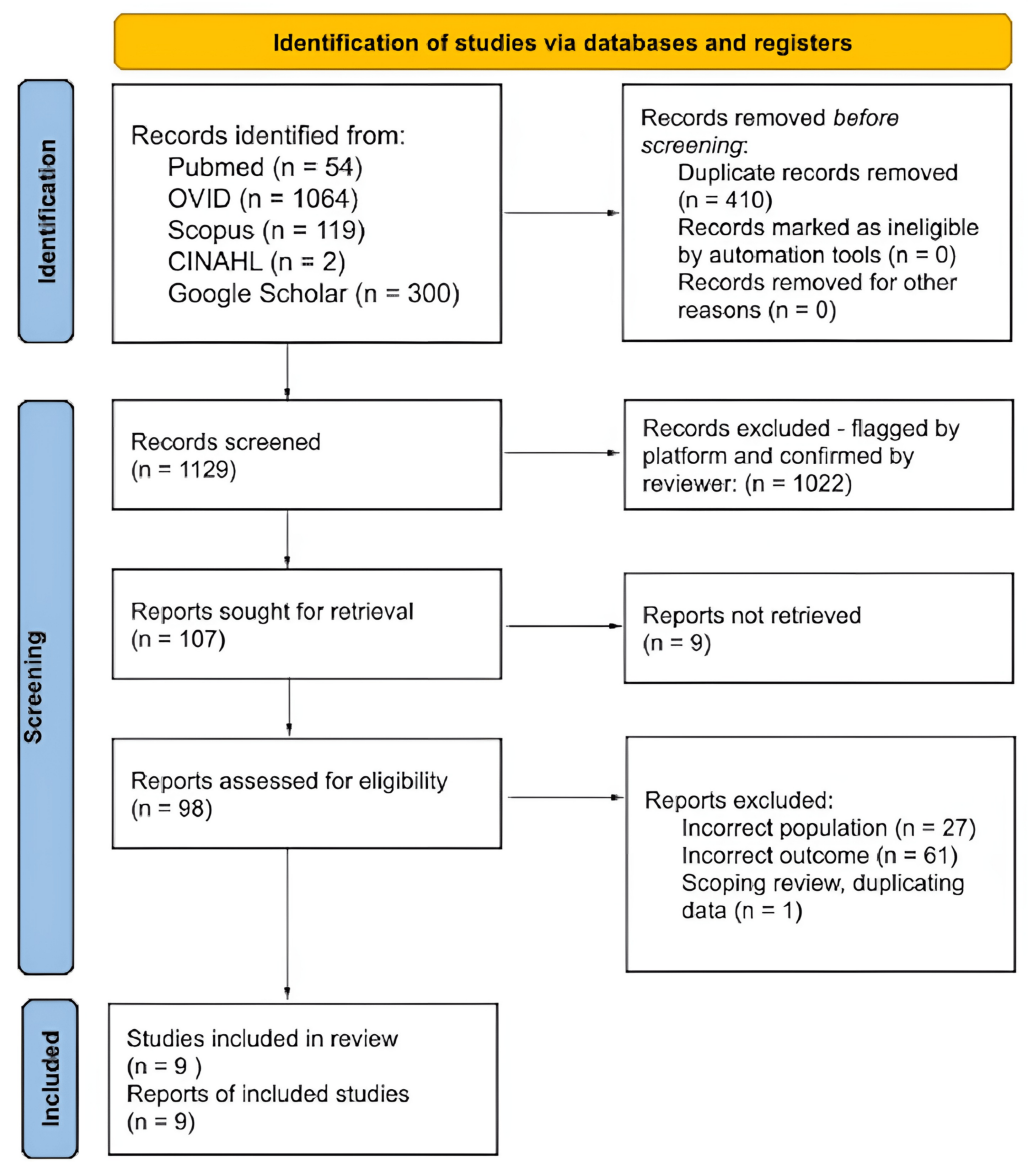
PRISMA sheet for the studies identified for review PRISMA: Preferred Reporting Items for Systematic Reviews and Meta-Analyses

Figure 1 displays the PRISMA flow diagram, outlining the study identification and screening process used in this systematic review [9]. The search encompassed 1129 records across all databases after duplicates were removed. Nine articles met the inclusion criteria for the analysis.

**Figure 2:**
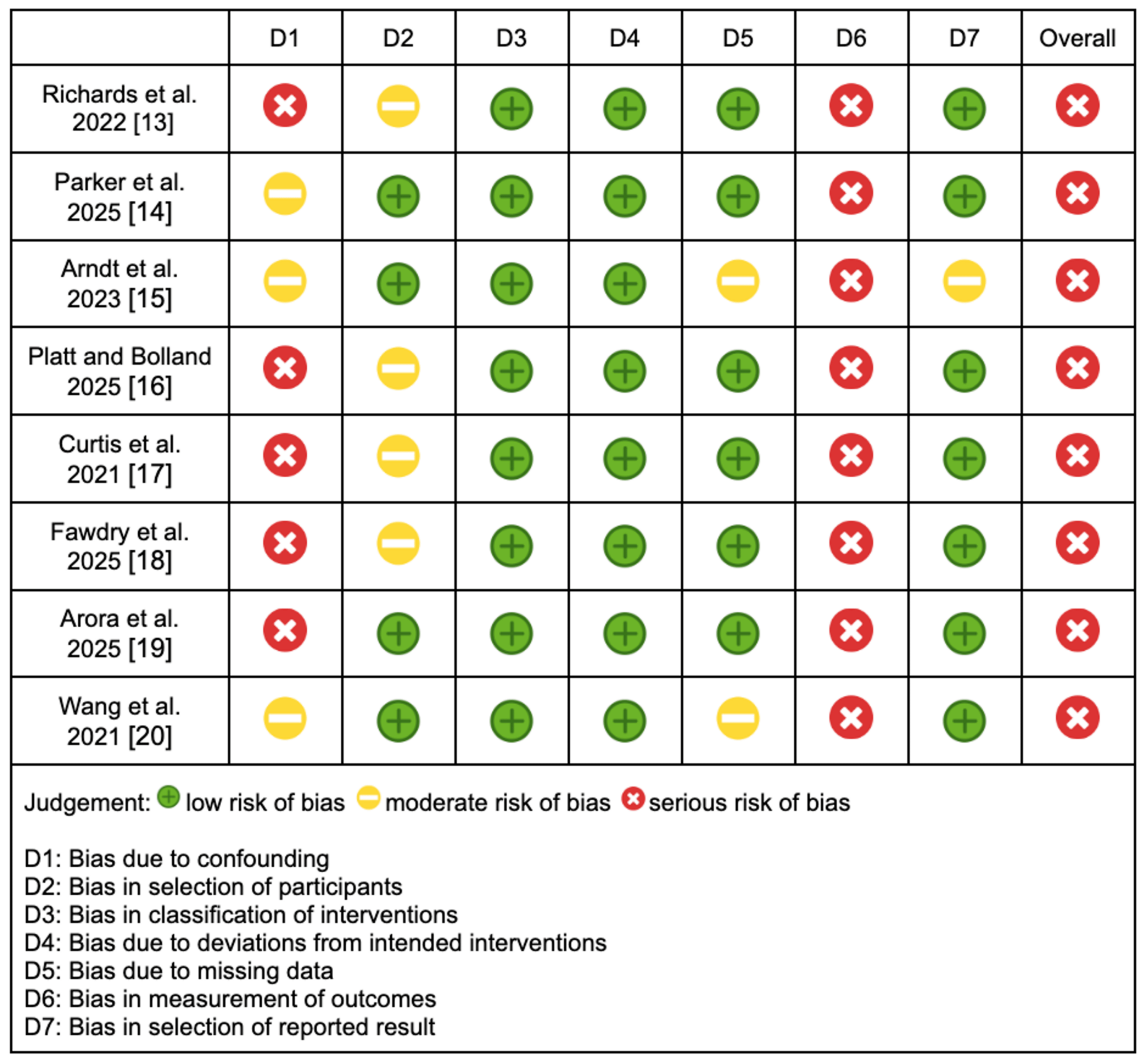
ROBINS-I tool for assessing risk of bias in non-randomised studies ROBINS-I: Risk of Bias in Non-randomised Studies of Interventions

Eight of the included studies were non-randomised retrospective cohort studies; therefore, the ROBINS-I tool was applied to assess quality and outcomes presented in Figure 2 [13-20]. A predominant source of bias arose from the measurement of outcomes across all eight studies, specifically related to the reliance on assumed travel data rather than direct measurement. This limitation resulted in a unanimous overall rating of serious bias for these non-randomised studies.

**Figure 3:**
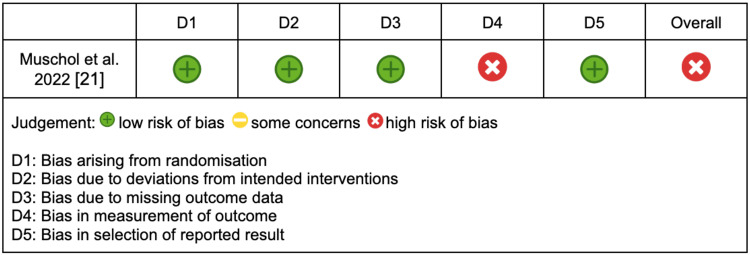
RoB 2 assessment for RCT RoB 2: Risk of Bias 2, RCT: randomised controlled trial

One study was an RCT and was assessed using the RoB 2 tool, with the results displayed in Figure 3 [21]. This study received an overall rating of high risk of bias, primarily driven by the same outcome measurement assumptions regarding patient travel and emissions calculations as in the non-randomised studies.

Table 2 summarises the characteristics and outcomes of the nine studies included. Studies were conducted between 2021 and 2025 in the United Kingdom (UK), the United States of America (USA), and Germany. The sample sizes varied significantly across the articles, as did the data sources and methods used to estimate emissions.

**Table 2:** Summary of findings

Author	Design	Sample size and setting	Intervention	Method to estimate emissions	Reduction in CO_2_e (kg)	Cost savings
Richards et al. 2022 [13]	Retroscpective cohort	52 - hip arthroplasty clinic - UK DGH	Telephone clinic	Travel, lighting, heating, waste vs nil	62.9	£7.18 for the patient, £162 per patient for the hospital
Parker et al. 2025 [14]	Cross-sectional study	508394 - general orthopaedics - USA large hospital and associated community clinics	Virtual - not explicitly specified	Travel, supplies, facility energy use vs technology energy use	7.08	Not reported
Arndt et al. 2023 [15]	Observational	51 - general orthopaedics - German University Hospital	Video	Travel vs power consumption of the data centre	11.8	Not reported
Platt and Bolland 2025 [16]	Retrospective analysis	319 - arthroplasty clinic - UK large hospital	Video - MS teams and telephone	Travel, heating, lighting, waste vs nil	65.2	Not reported
Curtis et al. 2021 [17]	Retrospective cohort	52 - trauma clinic - UK DGH	NF2F - not explicitly specified	Travel, heating, lighting, waste vs nil	59.1	£8.96 - 12 for the patient
Fawdry et al. 2025 [18]	Retrospective service review	107 - hand surgeon clinic - UK	Telephone	Travel vs nil	2.28	£1.84 - 2.80 for the patient
Arora et al. 2025 [19]	Retrospective review	416 - hand clinic - USA VA medical centre	Telephone	Travel vs nil	54.0	$30.69 for the patient
Wang et al. 2021 [20]	Retrospective cross-sectional analysis	298 - spinal clinic - USA hospital	Optimised telehealth - not explicitly specified	Travel, clinic facilities, telehealth infrastructure, laboratory energy vs the same	8.09	Not reported
Muschol et al. 2022 [21]	Prospective RCT	52 - general orthopaedics - German University Hospital	Video - digital health app	Travel vs nil	11.4	€9.53 for the patient

Reported Carbon Footprint Impact

All nine studies consistently reported a reduction in carbon footprint, predominantly attributed to decreased patient travel. The magnitude of this reduction, measured in kilograms of carbon dioxide equivalents (kgCO₂e), ranged from 2.28 to 65.2, with a median reduction of 11.8 kgCO₂e.

All studies derived emission data from estimates rather than direct measurement at the patient level. Emissions were derived from estimated patient travel distances using study-specific conversion factors.

Variability in Emissions Estimation Methods

A significant finding was the lack of consistency for converting patient travel to kgCO₂e, with no more than two studies using comparable methods. Two studies used the UK government GHG reporting conversion factors 2020, which estimate 0.28 kgCO₂e per mile [13,17]. Whilst another two used the Environmental Protection Agency GHG equivalences calculator, which estimates 0.411 kgCO₂e per mile in an average petrol car [14,19]. A further two used the computer programme TREMOD, developed by the German Federal Environment Agency, which assumes 0.143 kgCO₂e per kilometre in a car [15,21]. A single study converted average patient travel distance to kgCO2e using the Royal Automobile Club emission data, which, similar to the UK government, suggests a car produces 0.28 kgCO2e per mile [16]. The final two studies did not quote their conversion methods but used an online calculator or a life cycle assessment [18,20].

Assumptions regarding the mode of patient travel also differed. The majority of studies assumed that all patients travelled by car from their home address [13-16,19-21]. Only two studies deviated from this assumption; one surveyed patients to determine their actual travel modes (82% car, 18% other modes), whilst the other used Census data to estimate a probable 80% car-use rate [17-18].

Emission Sources Included

Five of the nine studies accounted for secondary emissions associated with clinic operations such as lighting, heating, and waste [13,14,16,17,20]. One study quoted 0.026 kgCO₂e per appointment, and another reported 0.004 kgCO₂e per appointment for their virtual platforms [14,15]. A single study described a life cycle assessment [20].

Certainty of Evidence

The certainty of evidence, assessed using the GRADE approach, was rated as low.

Reported Secondary Impacts

Five of the nine studies extended their analyses to secondary impacts, including patient time, cost savings, and accessibility benefits [13,17-19,21]. The primary benefit identified for patients was financial savings on petrol/fares and parking, followed by time saved from commuting and absence from work or school. Reported cost savings have been detailed in Table 2. These costs were not converted into a single currency to avoid introducing ambiguity from year-to-year exchange rate fluctuations. The certainty of the evidence for secondary impact data was low, as these outcomes were not directly measured in any study.

Subspecialities Analysis

The literature encompassed a variety of orthopaedic subspecialities, including general orthopaedics, trauma, hip arthroplasty, hand surgery, spinal surgery, and knee and shoulder surgery. No discernible correlation was found between the specific orthopaedic subspeciality studied and the magnitude of carbon footprint reduction achieved through the introduction of virtual care.

Discussion

Synthesising nine studies and encompassing 509,869 patients, the review demonstrated a median reduction of 11.8 kgCO₂e per patient, associated with a median travel distance of 24.6 miles. There was considerable variation in travel distance, ranging from 12 to 129.9 miles. Emission data was extrapolated from travel data using assorted conversion methods. This reliance on assumption introduced a notable risk of bias in the measurement of outcomes in both the ROBINS-I and RoB2 tools, as described in Figures 1-2. This introduced uncertainty regarding absolute effect estimates, as reflected by the low GRADE score.

These findings align with wider telemedicine literature. Van der Zee et al. conducted a systematic review assessing the carbon footprint of telemedicine across all medical specialities. Their overall conclusion that telemedicine reduces the healthcare carbon footprint by lowering patient travel aligns with and reinforces the findings of this review. They reported an average reduction of 25.6 kgCO₂e per consultation, primarily driven by reduced patient travel [6]. This figure is higher than the reductions observed in this review, which is consistent with their substantially greater median travel distance of 131 km per patient. Interestingly, in this review, the study that reported the lowest average patient travel distance also found the smallest reduction in carbon footprint with the introduction of telephone follow-up [18]. However, one study recorded considerably higher patient travel distances than the other studies, yet their reported kgCO₂e reduction was not an anomaly amongst the results [15]. This highlights the heterogeneity in emission calculation methods across the studies in this review.

Although patient travel was the dominant contributor to emissions savings, the methods used to estimate travel-related emissions were often simplistic. All included studies assumed petrol car travel and applied generic conversion factors, rather than capturing real-world driving patterns. The use of public transport should be considered more readily, and future work could also incorporate emissions measurement systems, routinely used in automotive testing, which more accurately quantify real-world emissions [22]. The increasing uptake of hybrid and electric vehicles may also reduce the relative benefit of telemedicine over time [23]. None of the studies accounted for evolving transport trends, and failure to include these variables limits the contemporary relevance and precision of their estimates.

The scope of emissions was often limited across the studies. Four studies did not consider secondary emissions associated with clinic operations such as lighting, heating, and waste. Emissions from virtual care were largely ignored, but when accounted for, their magnitude varied considerably. The unmeasured variables increased the risk of bias due to confounding in the ROBINS-I tool, which was depicted in Figure 2. The systematic review across all medical specialities also highlighted the omission of key variables in many primary studies, such as heating, lighting, and staff travel. It emphasised the need for streamlined life cycle assessments to strengthen the evidence base [6]. No study in this review reported whether clinicians conducted virtual clinics from home or from their workplace, and therefore, none incorporated staff commuting emissions into their calculations. Current practice suggests that most clinicians deliver virtual consultations on-site, meaning associated emissions remain unchanged. However, as digitalisation and flexible working become more widespread, remote clinic delivery may become feasible, potentially amplifying the carbon savings attributable to telemedicine. In the same vein, orthopaedic follow-up often requires obtaining a radiograph prior to the review. Patients may travel shorter distances for an X-ray than for a clinic, but the impact on the carbon footprint should be considered. Future analyses should capture these nuances to more accurately quantify the complete environmental impact of virtual care pathways.

The feasibility and equity of telemedicine warrant consideration. Orthopaedic outpatient populations include a large proportion of older adults, many of whom may have limited digital literacy or restricted access to appropriate technology. One study reported that patients unable to participate in video consultations were, on average, 17 years older than those who could [17]. This reinforces the need for structured triage systems to ensure only clinically appropriate and technologically capable patients are issued virtual appointments. The study conducted at a Veterans Affairs medical centre reported the greatest patient travel distance [19]. Excessive travel distance is a known barrier to accessing healthcare for many veterans, and reducing the need for travel provides significant accessibility benefits in this population [24].

Despite the limitations discussed, this study has methodological strengths that enhance the validity of its findings. It was conducted in accordance with a predefined protocol and adhered to PRISMA guidance, reducing bias in study selection and reporting. We used a structured PICO framework to run a broad, systematic search and capture all the necessary evidence. For reliability, two reviewers independently screened and extracted the key data. Only peer-reviewed primary studies were included, and established tools were used to appraise the risk of bias and certainty of evidence to provide a transparent evaluation of study quality.

Achieving net-zero healthcare emissions is a strategic priority for health systems worldwide. Outpatient services and exceptionally patient travel represent meaningful and modifiable contributors to the healthcare carbon footprint. While telemedicine is not a complete solution for net-zero healthcare, the consistent reduction in travel-related emissions demonstrated in this review supports its immediate implementation as a decarbonisation strategy. The findings highlight both the potential environmental benefits of virtual orthopaedic care and the areas that require refinement to achieve maximum future impact.

## Conclusions

Implementing or optimising existing virtual outpatient orthopaedic care pathways offers a solution that benefits both the planet and the patient. Although telemedicine is not a complete solution for net-zero healthcare, it consistently has a smaller carbon footprint than traditional in-person appointments, primarily by reducing patient travel. Patients also receive secondary benefits, including saving money on petrol and fares, taking less time away from work or school, and removing accessibility barriers for those who find long journeys challenging. The current research is consistent in its direction, but most available studies are observational, and the certainty of the evidence is low due to indirectness and assumptions in data collection. Therefore, further RCTs with standardised protocols and direct measurements of emissions are warranted to strengthen these findings.

## Appendices

**Table 3:** PICO Boolean search strings PICO: population, intervention, comparators, and outcomes

	Boolean search strings
P	(“orthopaedic” OR “orthopedic” OR “musculoskeletal” OR “fracture clinic” OR “arthroplasty clinic” OR “spine clinic” OR “sports medicine” OR “paediatric fracture” OR “rehabilitation”)
I	(“telemedicine” OR “telehealth” OR “virtual care” OR “virtual clinic” OR “remote consultation” OR “video consultation” OR “telephone consultation” OR “telerehabilitation” OR “e-consult”)
C	(“in-person” OR “face-to-face” OR “traditional clinic” OR “outpatient visit”)
O	(“carbon footprint” OR “greenhouse gas*” OR “CO2” OR “CO₂e” OR “emissions” OR “life cycle assessment” OR “environmental impact” OR “sustainability” OR “energy consumption” OR “travel distance”)

**Table 4:** Specific search strings used to extract articles for screening

Databases	Search String
MEDLINE, PubMed	((orthopedic[Title/Abstract] OR orthopaedic[Title/Abstract] OR trauma[Title/Abstract] OR fracture*[Title/Abstract] OR arthroplasty[Title/Abstract] OR "joint replacement"[Title/Abstract] OR spine[Title/Abstract])) AND ((outpatient*[Title/Abstract] OR "follow-up"[Title/Abstract] OR "ambulatory care"[Title/Abstract] OR clinic*[Title/Abstract])) AND ((telemedicine[Title/Abstract] OR telehealth[Title/Abstract] OR teleconsult*[Title/Abstract] OR virtual[Title/Abstract] OR remote[Title/Abstract] OR video[Title/Abstract] OR telephone[Title/Abstract] OR "digital health"[Title/Abstract])) AND (("carbon footprint"[Title/Abstract] OR "greenhouse gas*"[Title/Abstract] OR CO2[Title/Abstract] OR "carbon dioxide"[Title/Abstract] OR sustainability[Title/Abstract] OR "environmental impact"[Title/Abstract] OR "life cycle assessment"[Title/Abstract] OR LCA[Title/Abstract])) AND ("2008/01/01"[Date - Publication] : "3000"[Date - Publication])
Embase	(orthop* OR trauma OR fracture* OR arthroplasty OR "joint replacement" OR spine)/br AND (outpatient* OR "follow-up" OR "ambulatory care" OR clinic*) AND (telemedicine OR telehealth OR teleconsult* OR virtual OR remote OR video OR telephone OR "digital health") AND ("carbon footprint" OR "greenhouse gas*" OR CO2 OR "carbon dioxide" OR sustainability OR "environmental impact" OR "life cycle assessment" OR LCA) AND 2008-2026/py
Scopus, Web of Science, CINAHL, Google Scholar	(TITLE-ABS-KEY(orthop* OR orthopaedic OR trauma OR fracture* OR arthroplasty OR "joint replacement" OR spine)) AND TX ( TI orthop* OR TI orthopaedic OR TI trauma OR TI fracture* OR TI arthroplasty OR TI "joint replacement" OR TI spine ) AND TX ( TI outpatient* OR TI "follow-up" OR TI "ambulatory care" OR TI clinic* ) AND TX ( TI telemedicine OR TI telehealth OR TI teleconsult* OR TI virtual OR TI remote OR TI video OR TI telephone OR TI "digital health" ) AND TX ( TI "carbon footprint" OR TI "greenhouse gas*" OR TI CO2 OR TI "carbon dioxide" OR TI sustainability OR TI "environmental impact" OR TI "life cycle assessment" OR TI LCA ) Publication Date: 20080101-20261231 on 2025-09-27 07:21 PM ("orthopedic" OR "orthopaedic" OR trauma OR fracture* OR arthroplasty OR "joint replacement" OR spine) AND (outpatient* OR "follow-up" OR "ambulatory care" OR clinic*) AND (telemedicine OR telehealth OR teleconsult* OR virtual OR remote OR video OR telephone OR "digital health") AND ("carbon footprint" OR "greenhouse gas" OR CO2 OR "carbon dioxide" OR sustainability OR "environmental impact" OR "life cycle assessment" OR LCA)
